# Clozapine Monitoring in Older Adults: An Audit Evaluating Compliance With Clozapine Guidelines in Community Settings

**DOI:** 10.1192/bjo.2024.640

**Published:** 2024-08-01

**Authors:** Godwin Tong, Mella McCarthy

**Affiliations:** Hereford and Worcestershire Health and Care Trust, Worcester, United Kingdom

## Abstract

**Aims:**

To review compliance with current blood monitoring guidelines of Older Adult Community Mental Health (OACMHT) patients who are on clozapine within the community teams of Herefordshire and Worcestershire Health and Care NHS Trust. This is for full blood count, prolactin, glycated haemoglobin (HbA1C), liver function, renal function, lipid profile, glucose, and clozapine assay.

**Methods:**

Our trust guidelines state the following blood parameters should be monitored every 6 months:

1. Full Blood Count (FBC)
2. Glucose (fasting if possible)
3. Prolactin
4. Urea & electrolytes (U&E)
5. Lipid profile (fasting if possible)
6. Liver Function Tests (LFT)
7. HbA1c (annually)
8. Clozapine plasma assay (annually)

We reached out to the medical secretaries of the following OACMHTs: Wyre Forest, Malvern Evesham & Pershore, Worcester & Droitwich, Redditch & Bromsgrove to collate a list of patients on clozapine. We then retrospectively looked at blood test results in the past 1 year from 31.12.22 to 31.12.23 and assessed compliance of the 8 haematological parameters.

**Results:**

In total, 7 patients were identified across the 4 OACMHTs caseloads who were on clozapine. In the past 1 year, we would expect 2 episodes of monitoring for FBC, Glucose, U&E, Prolactin, Lipid profile, and LFT, as well as 1 episode of HbA1C and clozapine drug levels.

Compliance for FBC monitoring for 2 episodes was achieved for 100% (n = 7) of the patients. Compliance for 2 episodes of glucose and prolactin monitoring were 0%. Compliance for 2 episodes of renal profile monitoring was 57% (n = 4), but 86% (n = 6) of the patients had at least 1 episode of renal profile monitoring. Compliance for 2 episodes of Lipid profile monitoring was 0%, however 43% (n = 3) of the patients had at least 1 test. In terms of LFTs, 71% (n = 5) of the patients achieved the expected 2 episodes of monitoring, and 100% of them at least 1 episode of monitoring. For HbA1C monitoring, 100% of the patients had the expected 1 episode of monitoring annually. For clozapine plasma levels, 43% (n = 3) of the patients achieved their expected annual episode of monitoring.

An interesting observation of note was that a number of blood parameter investigations were performed by GPs/hospitals as part of another investigation, not exclusively for the sole purpose of clozapine monitoring. For example, 50% of the U&Es, 33% of lipid profiles, 71% of LFTs, and 43% of HbA1c tests were done by the GP/hospital.

**Conclusion:**

The OACMHTs within our trust achieved 100% compliance with FBC and HbA1c monitoring in the past 1 year. 71% compliance was achieved with LFT monitoring, 57% was achieved with U&E monitoring and 43% compliance was achieved with the annual clozapine monitoring.

With regards to tests done by GP/hospitals, on one hand, repeated phlebotomy of patients would come with increased direct medical (equipment, facilities) and non-medical (time) cost to service and intangible costs (pain) to patients. It would also not be cost effective to repeat these tests if done recently. Hence one could use recent test results as part of their monitoring routine. However, if these patients do not happen to see their GPs or have a hospital admission for unrelated issues, would they have missed their ideal monitoring targets? This unpredictability of timely monitoring raises the question of whether there is a need for the creation/standardisation of clozapine clinics within the OACMHTs, especially if the clozapine patient caseloads continue to grow.

